# Mitochondria Localize to the Cleavage Furrow in Mammalian Cytokinesis

**DOI:** 10.1371/journal.pone.0072886

**Published:** 2013-08-21

**Authors:** Elizabeth J. Lawrence, Craig A. Mandato

**Affiliations:** Department of Anatomy and Cell Biology, McGill University, Montreal, Quebec, Canada; Oregon Health & Science University, United States of America

## Abstract

Mitochondria are dynamic organelles with multiple cellular functions, including ATP production, calcium buffering, and lipid biosynthesis. Several studies have shown that mitochondrial positioning is regulated by the cytoskeleton during cell division in several eukaryotic systems. However, the distribution of mitochondria during mammalian cytokinesis and whether the distribution is regulated by the cytoskeleton has not been examined. Using live spinning disk confocal microscopy and quantitative analysis of mitochondrial fluorescence intensity, we demonstrate that mitochondria are recruited to the cleavage furrow during cytokinesis in HeLa cells. After anaphase onset, the mitochondria are recruited towards the site of cleavage furrow formation, where they remain enriched as the furrow ingresses and until cytokinesis completion. Furthermore, we show that recruitment of mitochondria to the furrow occurs in multiple mammalian cells lines as well as in monopolar, bipolar, and multipolar divisions, suggesting that the mechanism of recruitment is conserved and robust. Using inhibitors of cytoskeleton dynamics, we show that the microtubule cytoskeleton, but not actin, is required to transport mitochondria to the cleavage furrow. Thus, mitochondria are specifically recruited to the cleavage furrow in a microtubule-dependent manner during mammalian cytokinesis. Two possible reasons for this could be to localize mitochondrial function to the furrow to facilitate cytokinesis and / or ensure accurate mitochondrial inheritance.

## Introduction

Cytokinesis is the final stage of cell division in which one parent cell is physically divided into two daughter cells. In anaphase, astral and spindle microtubules specify the assembly of an actomyosin contractile ring at the equator of the cell between separating chromosomes [1]–[7]. This ensures that daughter cells inherit a proper complement of genetic and cytoplasmic material. As the actomyosin ring contracts, the plasma membrane at the equator of the cell pinches inwards, resulting in a cleavage furrow. The furrow continues to ingress until the cell is cleaved in two during abscission. A multitude of cellular and molecular processes take place at the furrow in order for cytokinesis to proceed, including actin polymerization, actomyosin contraction, signaling events, motor activity and membrane trafficking and fusion [8].

Mitochondria are the major producers of cellular ATP, which they produce through oxidative phosphorylation. In addition, mitochondria have been shown to maintain cellular redox state, regulate calcium homeostasis, synthesize membrane lipids and play important roles in cellular signaling pathways [9]. Mitochondria are dynamic organelles and, in higher eukaryotes, they are transported predominantly by the microtubule cytoskeleton, but can also anchor to actin [10]. The ability to translocate mitochondria to discrete subcellular regions permits the cell to spatially and temporally regulate mitochondrial activity in response to the metabolic needs of the cell. This is particularly evident in neurons, where trafficking of mitochondria to synaptic terminals is essential to meet the high energy and calcium-buffering requirements [10], [11]. In addition, intracellular calcium, ADP levels and nitric oxide have also been shown to influence mitochondrial motility, thereby constraining mitochondria to specific areas within the cell [12]–[15]. Cytokinesis is a metabolically demanding cellular event; however, whether mitochondria are specifically localized during cytokinesis is unknown.

Several studies have shown that mitochondrial distribution is regulated by the cytoskeleton during division in various eukaryotic cells [16]–[21]. Early work on scorpion spermatogenesis showed that mitochondria associate with microtubules at the mitotic spindle poles and are separated actively along with the chromosomes [16]. In fission yeast, mitochondria are delivered by microtubules to opposite cell poles during division to ensure faithful inheritance [17] and mitochondria in budding yeast are transported from mother to bud along actin cables [18], [19]. In the asymmetric division of *Acricotopus* germ line cells, mitochondria are preferentially segregated to one cell pole and may be involved in cell-fate determination [20]. Mitochondria have been shown to be uniformly distributed in dividing plant cells but then cluster transiently around the new cell wall at the site of division in a process requiring actin filaments [21]. When mammalian cells enter mitosis, the mitochondrial network is fragmented and is thought to remain randomly and passively dispersed throughout the cytoplasm until cytokinesis completion [22]. However, the distribution of mitochondria during mammalian cell division has not been examined.

Using spinning disk confocal microscopy, we examined the distribution of mitochondria during cytokinesis in cultured mammalian cells. Furthermore, using small molecule inhibitors of cytoskeleton dynamics, we investigated the role of actin and microtubules in determining mitochondrial positioning during division. Herein, we report for the first time that mitochondria are recruited to, and enriched at, the cleavage furrow during mammalian cytokinesis using a microtubule-dependent mechanism. Such microtubule-regulated positioning of mitochondrial activity at the cleavage furrow may have mechanistic implications in cytokinesis and mitochondrial inheritance.

## Materials and Methods

### Cell Culture and Drug Treatments

Mammalian cells were maintained at 37°C in a 5% CO_2_ incubator in culture media supplemented with 10% fetal bovine serum (FBS; Invitrogen) and 2 mM GlutaMAX (Gibco). HeLa, Ptk2 and Vero cells were grown in Minimum Essential Media (MEM; Invitrogen) and C2C12 cells were grown in Dulbecco’s Modiefied Eagles Medium (DMEM, Invitrogen). All cell lines were purchased from ATCC. To stain for mitochondria, live cells were incubated for 30 minutes with 40 nM MitoTracker Red CMX Ros or MitoTracker Green (Molecular Probes) in the dark. For the actin drug treatments, cells were treated for 15 minutes with media containing 0.1% DMSO (vehicle control), 100 nM Latrunculin A (Enzo Life Sciences) or 500 nM Jasplakinolide (Enzo Life Sciences) prior to imaging. For the microtubule drug treatments, media containing 0.1% DMSO (vehicle control), 20 μM Nocodazole (Calbiochem) or 10 μM Taxol (Sigma) was added to cells 2 or 4 minutes after anaphase onset. Cells with monopolar spindles were generated by incubation with 100 μM Monastrol for at least 2 hours but up to 12 hours (Enzo Life Sciences) and anaphase was induced by addition of 30 μM Purvalanol A (Enzo Life Sciences).

### Immunofluorescence

Cells on #1.5 coverslips were fixed with 3.2% paraformaldehyde in PBS for 15 minutes. Before antibody treatments, cells were permeabilized with PBS containing 0.1% Triton X-100 and blocked with 2% BSA in PBS for 30 minutes. Cells were incubated with primary antibodies overnight at 4°C and for 45 minutes at room temperature with secondary antibodies. The following primary antibodies were used: mouse anti-alpha-tubulin (Sigma, T9026), rabbit anti-GRP170 (a kind gift from J. Bergeron, McGill University, Montreal), mouse anti-golgin-97 (a kind gift from C. Morales, McGill University, Montreal) and anti-LAMP1 (Abcam, ab25630). Alexa Fluor 488-conjugated secondary antibodies were purchased from Invitrogen. F-Actin and DNA were stained with Phalloidin 488 (Invitrogen) and DAPI (Invitrogen) respectively.

### Microscopy and Imaging

Fixed images were acquired with a Confocor LSM 510 META confocal system on a Zeiss Axiovert 200 M inverted microscope using a 1.4 NA 63x oil-immersion objective and Zen imaging software (Zeiss). For live imaging, HeLa cells were grown on 35 mm glass-bottom dishes (1.5 mm thickness; MatTek) and maintained on the microscope stage in phenol-free MEM media (Invitrogen) at 37°C and 5% CO_2_ using a Chalamide TC environmental control system (Live Cell Instruments). All live imaging was performed using a Quorum WaveFX-X1 spinning disk confocal system on a Leica DMI6000B inverted microscope (Quorum Technologies Inc.). Images were acquired using a 1.4 NA 63x oil-immersion objective and captured with a Hamamatsu ImagEM EM-CCD camera controlled with Metamorph software (Molecular Devices). MitoTracker Red fluorescence was excited with a 568 nm laser and 50 ms exposure time and collected with an ET 620/60 emission filter set. MitoTracker Green fluorescence was excited with a 491 nm laser and 50 ms exposure time and collected with a ET 525/50 emission filter set. For time-lapse experiments, images were collected every minute. At each time point, 20 z-series optical slices were obtained with a step size of 1.0 μm using an ASI MS-2000 piezo stage. The brightness, contrast and background were adjusted in Metamorph for presentation purposes only.

### Image and Statistical Analysis

To quantify changes in mitochondrial distribution during mitosis, the average fluorescence intensity from cell pole to equator was measured using images of cells in five representative stages of cell division from metaphase to late cytokinesis. The length of cell division was normalized to account for variations between cells. The start and end points of division were defined by eye from DIC images, with t = 0 being defined as one time point prior to anaphase onset and t = 1 indicating a fully ingressed cleavage furrow. The five representative division stages were extracted from the normalized time series as follows: metaphase (t = 0), anaphase (t = 0.25), early-cytokinesis (t = 0.50), mid-cytokinesis (t = 0.75), late-cytokinesis (t = 1). For cells in which cytokinesis was inhibited, the length of division was taken to be the same as the average division length of control cells and the representative time points were selected as described above. The average fluorescence intensity along the entire perimeter of the cell at each stage of division was measured by performing a linescan at a distance of 15 pixels from the edge of the cell. To generate the linescan, the perimeter of the cell in the DIC image was manually traced and the area outside of the cell was painted black. The image was then thresholded and binerized in order to generate a Euclidean distance map. Based on this distance map, the image was thresholded at a distance of 15 pixels from the edge of the cell and a region was created around the threshold. The region was then transferred to the corresponding fluorescence image and used to perform a 15 pixel-wide linescan of the average fluorescence intensity. The distance, average pixel intensities and four reference-coordinates corresponding to the two poles and two furrows were logged in Excel. In order to average data across several cells, the pole-to-furrow distance was normalized and the fluorescence intensities were binned and then normalized relative to the mean intensity of the data set. The data used for quantification was obtained in Metamorph using background-corrected, but otherwise unaltered, images from live imaging experiments. Data were normalized and averaged in MATLAB (Mathworks) then transferred to GraphPad (Prism) for graph plotting and statistical analysis. Lines were fitted to the data using nonlinear regression and statistical significance was assessed using the extra-sum-of-squares F-Test (α = 0.05). To assess the symmetry of mitochondrial inheritance, the total mitochondrial fluorescence intensity in pairs of daughter cells was measured by summing the fluorescence intensities from each z-stack slice in the region corresponding to each daughter cell, then dividing the two summations to give an inheritance ratio, where 1 = equal inheritance and 0 =  unequal inheritance (Metamorph). Data were plotted as a box and whisker graph in GraphPad (Prism) and statistical significance was assessed using the Student’s T-Test (α = 0.05).

## Results

### Mitochondria localize to the cleavage furrow and are depleted at the cell poles in dividing mammalian cells

**Figure 1:**
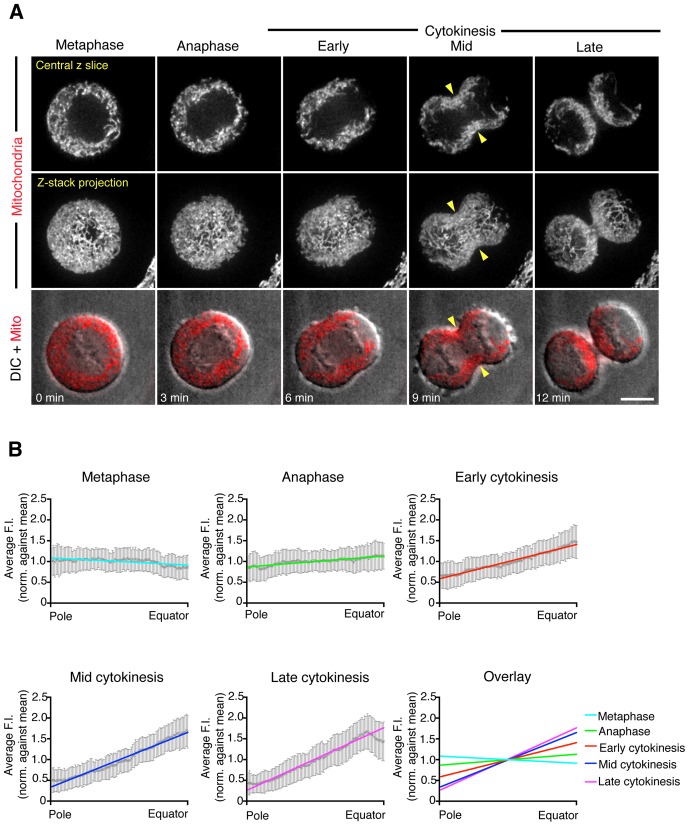
Mitochondria localize to the cytokinetic cleavage furrow in dividing HeLa cells. (A) Spinning disk confocal time-lapse images of HeLa cells stained with 40 nM MitoTracker Red to visualize mitochondria. The time points are representative of five stages of division from metaphase to late-cytokinesis. Shown are the following: a single focal plane from the center of the confocal stack (upper row), a maximum projection of the full Z-stack (middle row) and a merge of the DIC image with the mitochondrial single focal plane (bottom row). The full time-lapse can be seen in Movie S1. Note the enrichment of mitochondria in the region of the cleavage furrow and reduction at the cell poles as division progresses. Yellow arrowheads indicate the position of the cleavage furrow. Time is given in minutes after anaphase onset. Bar, 10 µm. (B) Quantification of the distribution of mitochondria from cell pole to equator at each representative stage of division. An overlay of all five stages is also shown (last panel). The normalized distance from cell pole to equator is displayed on the x-axis and the average mitochondrial fluorescence intensity is displayed on the y-axis. Data are represented as mean +/– SEM (25 cells, N = 100) and lines fitted by non-linear regression.

**Figure 2:**
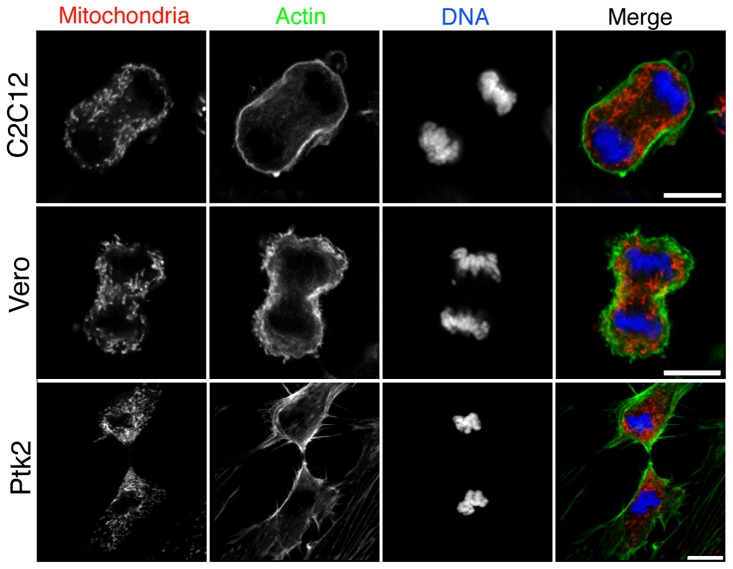
Mitochondria localize to the cleavage furrow in C2C12, Vero and Ptk2 cells. Fixed images of dividing C2C12, Vero and Ptk2 cells in early cytokinesis (C2C12), mid cytokinesis (Vero) and late cytokinesis (Ptk2). Cells were co-stained for mitochondria (MitoTracker Red, red), actin (Phallodin-488, green) and DNA (DAPI, blue). Bar, 10 µm.

To characterize the distribution of mitochondria from metaphase to late cytokinesis, we performed live imaging of dividing HeLa cells stained with MitoTracker Red to visualize mitochondria and quantified the mitochondrial fluorescence intensity from cell pole to equator (Figure 1). Representative images of five stages of mitosis from metaphase to late cytokinesis are shown in Figure 1A and the complete time-series can be viewed in Movie S1. The overlay of the mitochondrial signal with DIC images (Figure 1A, bottom row) allowed the distribution of the mitochondria to be examined in relation to the chromosomes and cytokinetic cleavage furrow. In metaphase, the mitochondrial population was evenly distributed in the cytoplasm around the metaphase plate. During anaphase, the mitochondria began to accumulate at the cell equator, the future site of the cytokinetic cleavage furrow, and were depleted from the cell poles. As the cleavage furrow formed in early cytokinesis, the mitochondria were further enriched at the furrow and reduced at the cell poles. In mid-cytokinesis, the mitochondrial population was strongly polarized towards the cleavage furrow, assuming a symmetrical arrangement around the furrow. The polarization of mitochondria towards the cleavage furrow was maintained until late cytokinesis. These observations were confirmed by quantifying the average fluorescence intensity from cell pole to equator at each of the five representative division stages (Figure 1B). The polarization of the mitochondria from pole to equator was found to be statistically significant in anaphase compared with metaphase and remained significant throughout the subsequent stages of division (F-Test, p<0.05). The strongest polarization of mitochondria was observed in late cytokinesis (Figure 1B, late cytokinesis). Note that the dip in intensity at the furrow in late cytokinesis is due to exclusion of the mitochondria from the densely bundled mid-zone microtubules. We observed the same polarization of mitochondria in cells stained with MitoTracker Green, which stains mitochondria in proportion to mass and independently of mitochondrial membrane potential (Figure S1). Additionally, we found mitochondria enriched at the cleavage furrow in a variety of other mammalian cell lines including mouse myoblasts (C2C12), African monkey kidney epithelial cells (Vero) and rat kangaroo kidney epithelial cells (Ptk2) (Figure 2).

### Mitochondrial distribution in cytokinesis is distinct from the endoplasmic reticulum (ER), Golgi and lysosomes

**Figure 3:**
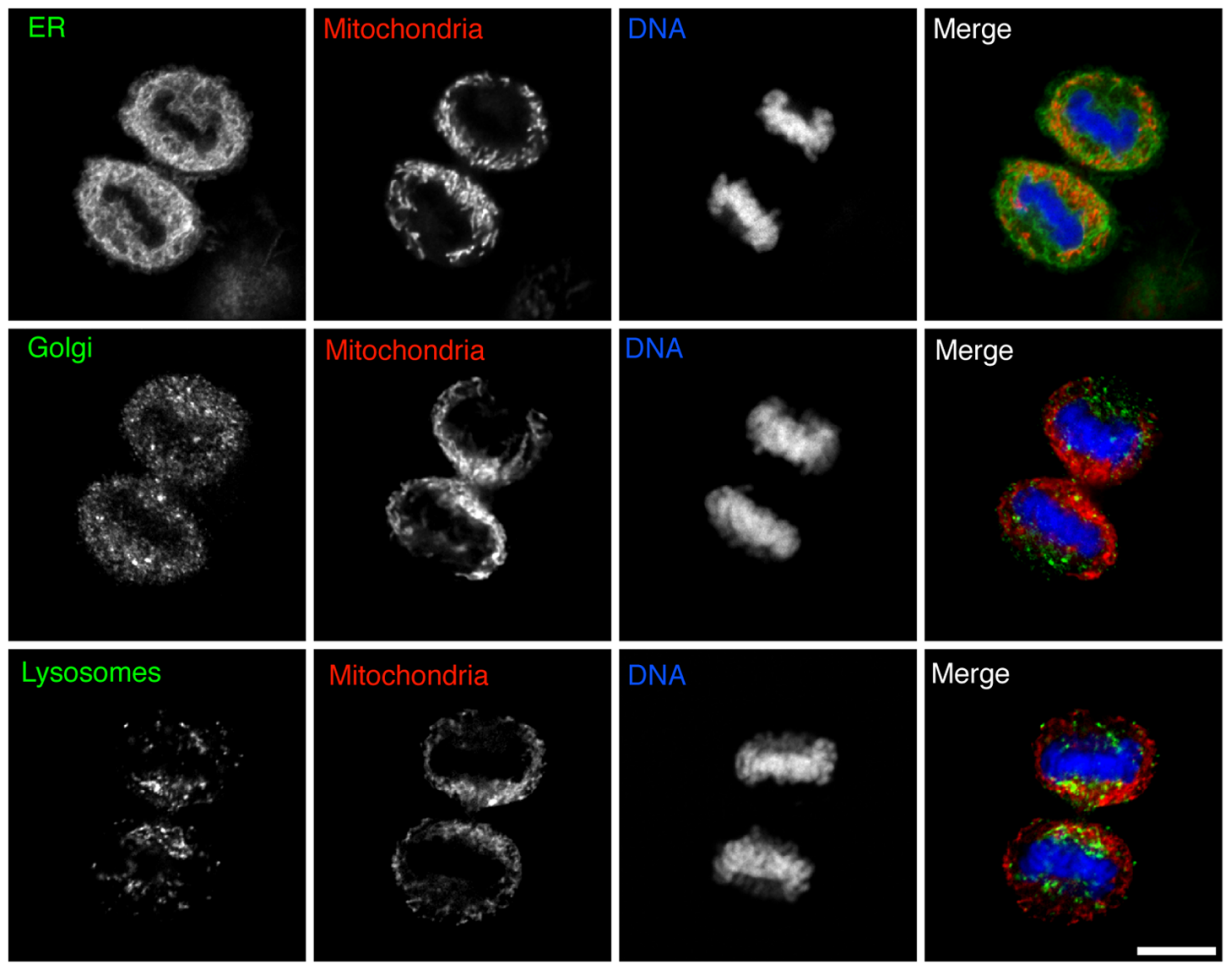
Mitochondrial localization in cytokinesis is distinct from the endoplasmic reticulum, Golgi and lysosomes. Fixed images of HeLa cells in late cytokinesis co-stained for mitochondria (MitoTracker Red, red), DNA (DAPI, blue) and endoplasmic reticulum (anti-GRP-170), Golgi (anti-Golgin-97) or lysosomes (anti-Lamp-1) shown in green. Bar, 10 µm.

We compared the localization of the ER, Golgi and lysosomes with mitochondria in late cytokinesis by immunofluorescence (Figure 3). HeLa cells were stained for mitochondria (MitoTracker Red) then fixed and co-stained for the ER (anti-GRP-170), Golgi (anti-Golgin-97) or lysosomes (anti-Lamp-1) and DNA (DAPI). Consistent with our previous results, the mitochondria were enriched at the cleavage furrow and reduced at the cell poles. In contrast to mitochondria, the ER was distributed throughout the cytoplasm excepting the areas occupied by chromosomes (Figure 3, top row). We did, however, notice a slight enrichment of the ER at the furrow, which may be explained by the tight association between the ER and mitochondria [23], [24]. The Golgi-derived membranes were dispersed throughout the cytoplasm but were enriched in the region of the spindle poles (Figure 3, middle row). Finally, the lysosomes were localized predominantly in the region of the bundled mid-zone microtubules and the spindle poles (Figure 3, bottom row). The distinct localization of mitochondria at the cleavage furrow suggests that mitochondria are localized by a different mechanism than the ER, Golgi and lysosomes.

### Mitochondria localize to the cleavage furrow during multipolar and monopolar divisions

**Figure 4:**
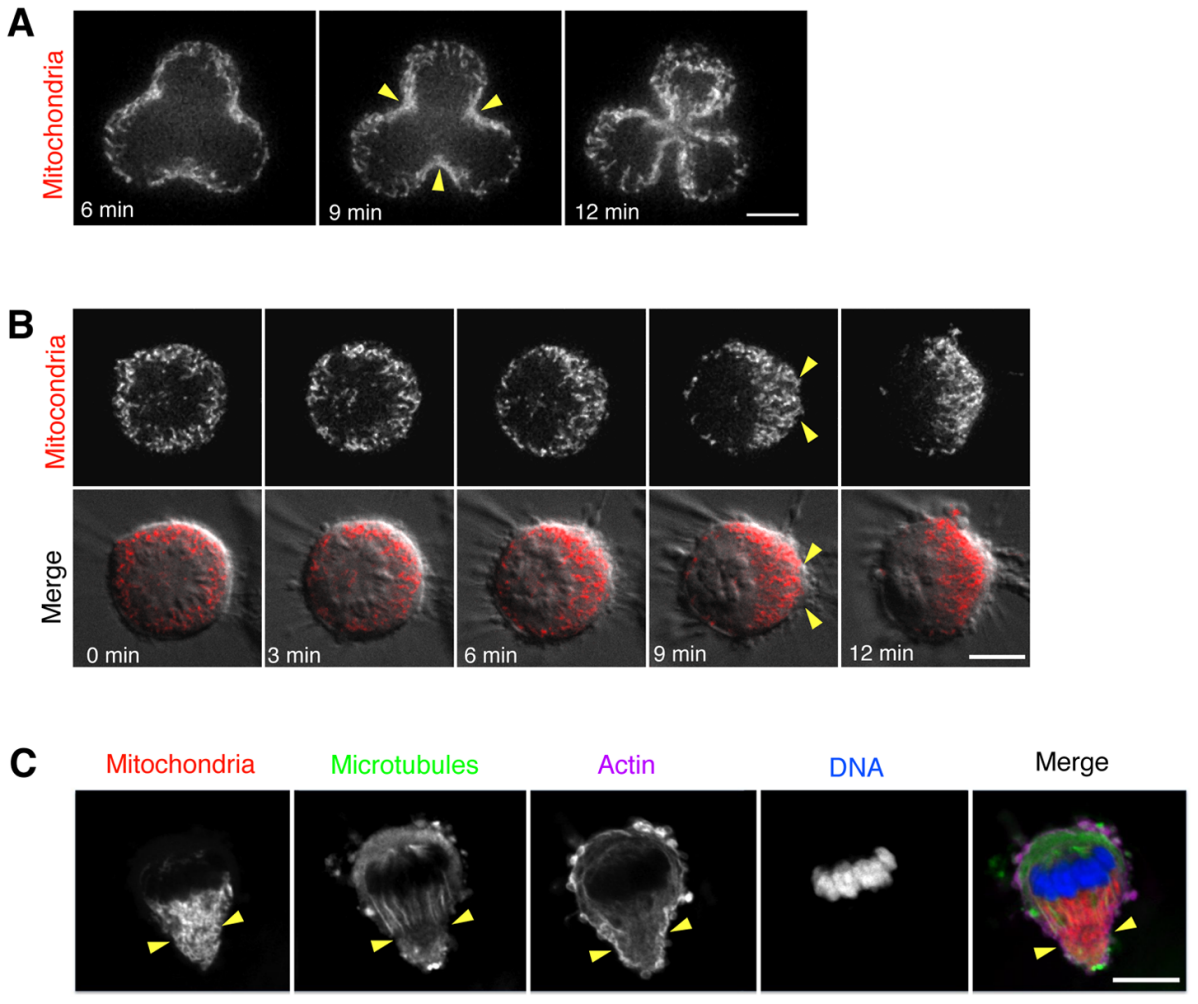
Mitochondria localize to the cleavage furrows in multipolar and monopolar cytokinesis. (A) Live-imaging of an aberrant HeLa cell with a tripolar spindle undergoing cytokinesis stained with MitoTracker Red to visualize mitochondria. Three frames are shown from early to late cytokinesis. (B) Live-imaging of HeLa cells stained with MitoTracker Red that were treated with Monastrol to generate monopolar spindles then induced into cytokinesis with Purvalanol A. The time points shown are representative of five stages of monopolar division. The full time-lapse can be seen in Movie S2. Time is given in minutes after the first visible sign of chromosome polarization (indicating the onset of monopolar division). (C) Same as (B), but cells were fixed and stained for mitochondria (MitoTracker Red, red), microtubules (anti-alpha-tubulin, green), actin (Phalloidin-488, magenta) and DNA (DAPI, blue). Yellow arrowheads indicate the position of the cleavage furrow. Bar, 10 µm.

In cultured HeLa cells, we occasionally observed aberrant tripolar and tetrapolar mitotic cells. In such multipolar divisions, the mitochondria accumulated at each of the multiple cleavage furrows (Figure 4A). In light of this observation, we decided to test whether mitochondria would also localize to the cleavage furrow during monopolar cytokinesis in which cytokinesis is forced to occur independently of nuclear division. Monastrol is an Eg5 inhibitor that prevents centrosome separation [25]. Cells were treated with Monastrol to cause arrest in mitosis with monopolar spindles and were then induced into cytokinesis by inhibition of Cdk1 with Purvalanol A (Figure 4B and Movie S2) [26], [27]. Prior to the addition of Purvalanol A, the mitochondria were evenly distributed throughout the cytoplasm (Figure 4B, 0 min). Addition of Purvalanol A caused the cells to undergo a type of polarized cytokinesis in which the chromosomes moved to one side of the cell but did not separate and a cytoplasmic protrusion and cytokinetic cleavage furrow formed at the opposite side of the cell (Figure 4B, 3–12 min). Consistent with our observations in normal bipolar division (Figure 1), the mitochondria were progressively polarized towards the monopolar cleavage furrow (Figure 4B, yellow arrows). Collectively, the data suggest a robust mechanism, which actively and specifically recruits mitochondria to the cleavage furrow in cytokinesis independently of nuclear division.

To visualize the cytoskeleton during monopolar cell division using immunofluorescence, live Monastrol-treated cells were incubated with MitoTracker Red to stain mitochondria, then fixed at various time points prior to or following Purvalanol A addition. Cells were then stained for microtubules (anti-alpha-tubulin), actin (phallodin-488) and DNA (DAPI). An image of a representative cell in mid-cytokinesis is given in Figure 4C. We observed that mitochondria localized in the direction of the highly polarized microtubules as well as in proximity to regions of actin enrichment (Figure 4C, yellow arrowheads). These observations suggest that the mechanism of mitochondrial recruitment may be mediated by the actin and microtubule cytoskeletons.

### The actin cytoskeleton is not required for the recruitment or anchoring of mitochondria to the cleavage furrow

**Figure 5:**
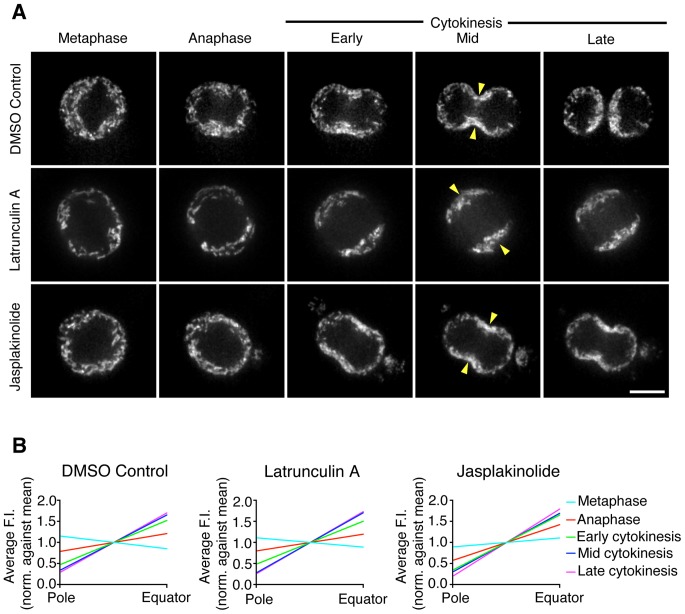
Mitochondrial recruitment to the cleavage furrow is not dependent on F-actin. (A) Live-imaging of HeLa cells treated with DMSO (upper row), Latrunculin A (middle row) or Jasplakinolide (bottom row) and stained with MitoTracker Red to visualize mitochondria. Five representative frames are shown from metaphase to late cytokinesis. The full time-lapse can be seen in Movie S3. Yellow arrowheads indicate the position of the cleavage furrow. Bar, 10 µm. (B) Quantification of the distribution of mitochondria from cell pole to equator at each stage of division in cells treated with DMSO (left panel; 11 cells, N = 44), Latrunculin A (middle panel; 8 cells, N = 32) and Jasplakinolide (right panel; 8 cells, N = 32). The normalized distance from cell pole to equator is displayed on the x-axis and the average mitochondrial fluorescence intensity is displayed on the y-axis. Data are represented as the mean and lines fitted by non-linear regression (see also Figure S3).

In Figure 1 we observed that mitochondria localized towards the cell cortex in the region of the cytokinetic cleavage furrow. The cortex and cleavage furrow are both actin-rich structures and, in accordance with this, we showed that mitochondria localized to the actin-rich furrow in monopolar cytokinesis (Figure 4C). Therefore, using inhibitors of actin dynamics, we tested whether actin was required for the recruitment or docking of mitochondria to the cleavage furrow (Figure 5 and Movie S3). Cells were treated with either DMSO as a control or Latrunculin or Jasplakinolide to depolymerize or stabilize actin respectively (Figure S2, top row, and Figure S3) [28]–[30]. As expected, mitochondria were recruited to the cleavage furrow and away from the cell poles in DMSO-treated control cells (Figure 5A, top row, and Figure 5B, left panel). When actin was depolymerized with Latrunculin A, nuclear division proceeded normally but contractile ring formation and cytokinesis were inhibited. We observed that mitochondria were recruited towards the equator of the cell and away from the cell poles despite the absence of actin filaments (Figure 5A, middle row). Quantification of mitochondrial fluorescence from pole to equator (Figure 5B, middle panel) showed that the mitochondrial distribution in Latrunculin-treated cells was not significantly different from control cells for all stages of division (F-Test, p>0.05). In the presence of Jasplakinolide, nuclear division and contractile ring formation proceeded normally but furrow ingression was inhibited due to the stabilization of actin filaments. Following Jasplakinolide treatment, mitochondria were recruited to the equator and away from the cell poles despite the enrichment of stabilized actin (Figure 5A, bottom row). Quantification confirmed that the mitochondrial distribution in Jasplakinolide-treated cells (Figure 5B, right panel) was not significantly different from control cells (F-Test p>0.05). Therefore, these results indicate that the recruitment of mitochondria towards the cell equator is not dependent on actin.

### Mitochondria are transported on microtubules to the cleavage furrow

**Figure 6:**
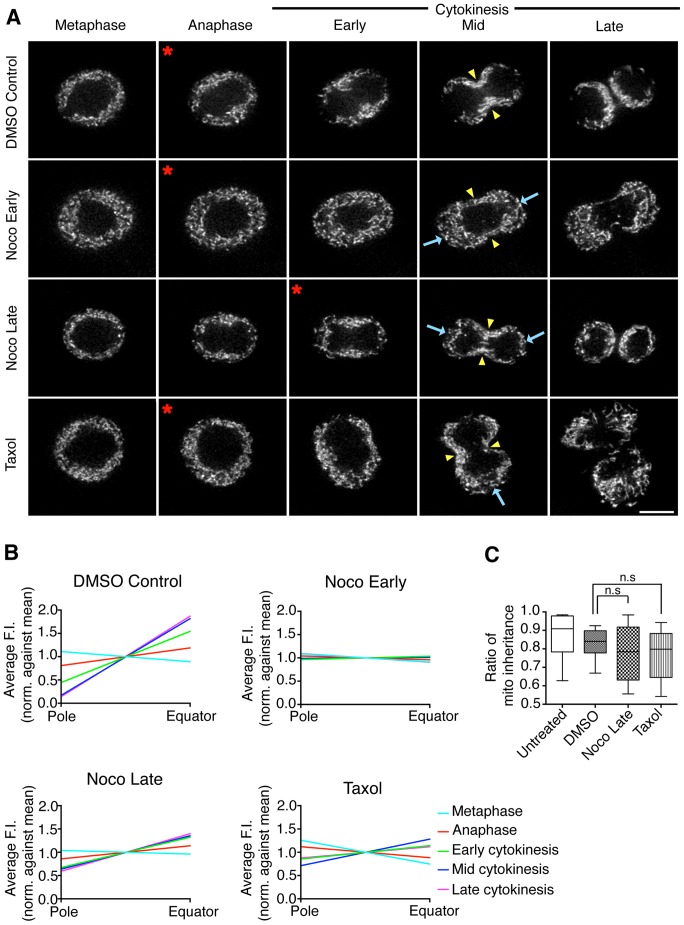
Mitochondrial recruitment to the cleavage furrow is dependent on microtubules. (A) Live-imaging of HeLa cells treated with DMSO (top row), Early Nocodazole (second row), Late Nocodazole (third row) or Taxol (bottom row) and stained with MitoTracker Red to visualize mitochondria. Five representative frames are shown from metaphase to late cytokinesis. Treatments were added to live cells either 2 (DMSO, Noco Early, Tax) or 4 minutes (Noco Late) after anaphase onset. Note the mislocalized mitochondria at the cell poles (blue arrows) in drug-treated cells compared with DMSO-treated control cells. Red asterisks indicate the first time point following drug-addition and yellow arrowheads indicate the position of the cleavage furrow. The full time-lapse can be seen in Movie S4. Bar, 10 µm. (B) Quantification of the distribution of mitochondria from cell pole to equator at each stage of division in cells treated with DMSO (top left; 8 cells, N = 32), Early Nocodazole (top right; 8 cells, N = 32), Late Nocodazole (bottom left; 10 cells, N = 40) and Taxol (bottom right; 6 cells, N = 24). The normalized distance from cell pole to equator is displayed on the x-axis and the average mitochondrial fluorescence intensity is displayed on the y-axis. Data are represented as the mean and lines fitted by non-linear regression (see also Figure S4). (C) Quantification of mitochondrial inheritance by daughter cells in control (Untreated and DMSO) and drug-treated (Noco Late and Taxol) cells. Data are represented by a box and whisker plot showing maximum, minimum, upper and lower quartiles and sample median. n.s  =  not significant.

Microtubules are required to establish the site of furrow formation in mammalian cytokinesis [7]. In addition, mitochondrial transport in non-dividing cells is mediated by microtubules [31]. Furthermore, as noted above, we observed mitochondrial enrichment in the direction of the plus-ends of the polarized microtubules in monopolar cytokinesis (Figure 4C). Therefore, using inhibitors of microtubule dynamics, we tested whether the transport of mitochondria to the cleavage furrow was microtubule-dependent (Figure 6 and Movie S4). Cells were treated with either DMSO as a control or Nocodazole or Taxol to depolymerize or stabilize microtubules respectively (Figure S2, bottom row and Figure S4) [32]–[34]. Note that after 5 minutes of Taxol-treatment the appearance of interphase microtubules were not visibly altered (Figure S2, bottom row, last panel). However, Taxol-treatment of dividing cells caused inhibition of chromosome separation indicating that microtubules were effectively stabilized (data not shown).

DMSO-treated control cells displayed normal recruitment of mitochondria to the cleavage furrow and away from the cells poles (Figure 6A, top row and Figure 6B, top left panel). When microtubules were depolymerized with Nocodazole in early anaphase, nuclear division, contractile ring formation and cytokinesis were inhibited. The mitochondria remained homogenously distributed in the cytoplasm and were not enriched at the cell equator or reduced at the poles (Figure 6A, second row). This observation was confirmed by quantification (Figure 6B, top right panel), which showed that there was no significant polarization of mitochondria in early Nocodazole-treated cells throughout division (F-Test, p>0.05). Microtubules specify cytokinesis in anaphase; therefore, to quantify mitochondrial distribution in cells depleted of microtubules but which are able to undergo cytokinesis, Nocodazole was added after cytokinesis specification in late anaphase (Figure 6A, third row). In late Nocodazole-treated cells, some mitochondria had already begun to redistribute away from the poles and towards the furrow at the time of drug addition; however, further mitochondrial recruitment was inhibited upon addition of Nocodazole and depolymerization of microtubules (Figure 6A, third row). Quantification of late Nocodazole cells (Figure 6B, bottom left panel) showed that the mitochondrial distribution was significantly less polarized compared with control cells from early cytokinesis onwards, consistent with the addition of the drug in late anaphase (F-Test, p<0.05). Furthermore, there was no significant change in the mitochondrial distribution in late Nocodazole cells from early cytokinesis until the end of division ((F-Test, p>0.05). Together, these results indicate that microtubules are required to polarize mitochondria towards the cleavage furrow during cytokinesis.

When microtubules were stabilized with Taxol in early anaphase, nuclear division was inhibited but cytokinesis proceeded. The mitochondria remained homogenously distributed throughout the cytoplasm until early cytokinesis (Figure 6A, bottom row) when some polarization of mitochondria was observed. Quantification indicated that mitochondria polarized at a later stage in Taxol-treated cells compared with control cells (Figure 6B, bottom right panel). Specifically, the polarization of mitochondria in Taxol-treated cells became significant in early cytokinesis compared to anaphase in control cells (F-Test, p<0.05). Furthermore, the degree of polarization was significantly lower in Taxol-treated cells compared with controls (F-Test, p<0.05). Since Taxol-treatment stabilizes microtubules and abolishes microtubules dynamics, this suggests that microtubule dynamics may be involved in the localization of mitochondria to the furrow.

To determine whether microtubules were required for accurate mitochondrial inheritance, we measured the total mitochondrial fluorescence in pairs of daughter cells and expressed the symmetry of inheritance as a ratio where 1 =  equal inheritance and 0 =  unequal inheritance (Figure 6C). Untreated and DMSO-treated control cells had median inheritance ratios of 0.91 (N = 5) and 0.85 (N = 8) respectively, indicating equal inheritance. In comparison, Nocodazole-treated cells had a median ratio of 0.78 (N = 10) and Taxol-treated cells had a median ratio of 0.79 (N = 6) indicating a tendency towards unequal inheritance compared with control cells, although this was not statistically significant (T-Test, p>0.05). Furthermore, both Nocodazole- (IQR = 0.29) and Taxol- (IQR = 0.23) treated cells showed a larger spread of inheritance ratios compared with DMSO control cells (IQR = 0.12), suggesting that disruption of microtubules reduced the accuracy of mitochondrial partitioning.

## Discussion

Mitochondria are essential for cellular function; as such, mitochondria must be precisely positioned within the cell and accurately segregated into daughter cells during division. Herein, we report that mitochondria are recruited to the cleavage furrow during mammalian cytokinesis (Figure 1). Furthermore, this localization is distinct from the ER, Golgi and lysosomes (Figure 3). Consistent with this, mitochondrial localization to the cleavage furrow has been noted at least twice previously in other somatic mammalian cells [27], [35], as well as in the first division of porcine oocytes [36] and in grasshopper spermatocytes [37]. In addition, we observed mitochondrial recruitment to the cleavage furrow in multiple mammalian cell lines (Figure 2) and in aberrant monopolar and multipolar divisions (Figure 4), suggesting that the mechanism of recruitment to the furrow is robust and conserved in mammals.

In higher eukaryotes, long-range mitochondrial transport is dependent on microtubules, while short-range movements and anchoring are mediated by actin [31], [38]. We demonstrated with pharmacological interference of the cytoskeleton that the localization of the mitochondria to the cleavage furrow is dependent on microtubules (Figure 6) but not on actin (Figure 5). We saw no effect on mitochondrial recruitment or localization to the cleavage furrow when actin was depolymerized or stabilized (Figure 5). This is surprising considering the importance of actin-mediated movements in various systems including budding yeast, in which actin cables are the primary cytoskeleton track for mitochondrial transport [18], [19], and in neurons, where mitochondria dock to actin in synaptic terminals and growth cones [39], [40]. In addition, a novel human Myosin 19 that functions in actin-based mitochondrial movements has recently been identified [41]. Therefore, we cannot rule out a possible role for actin in mediating short-range, local movements of mitochondria following their delivery to the cleavage furrow which may not be detectable using the quantitation method developed in this study. Previous studies have shown that the distributions of other organelles during mammalian cell division are mediated by microtubules of the centrosomes and the mitotic spindle [42]–[45]. We observed that mitochondria accumulated at the cleavage furrow in the region of equatorial astral microtubules and in the direction of the plus-ends of polarized microtubules at the cleavage furrow during monopolar division (Figure 4) [27]. This raised the possibility that the equatorial astral microtubules may be involved in trafficking mitochondria to the furrow. Indeed, depolymerization of microtubules with Nocodazole inhibited the recruitment of mitochondria to the furrow (Figure 6). When microtubules were stabilized with Taxol, the polarization of the mitochondria towards the cleavage furrow occurred at a later stage and was significantly reduced compared with controls (Figure 6). During division, astral microtubules are important for cleavage furrow induction and spindle positioning [2], [4]. In dividing cells, not all astral microtubules are equal; a subset of astral microtubules which contact the cortex near the furrow are selectively stabilized, while those at the cell poles are more dynamic [6], [7]. The more stable equatorial astral microtubules are thought to make effective tracks for delivering factors required for cytokinesis to the furrow [46]. It is conceivable that, following Taxol-treatment, the normally dynamic astral microtubules at the cell poles are stabilized and, consequently, become available for use as alternative tracks for mitochondrial transport. This would explain why mitochondria are retained at the cell poles in Taxol-treated cells and polarization towards the furrow is reduced. To our knowledge, this is both the first time that astral microtubules have been implicated in regulating organelle positioning during division and that mitochondria have been shown to have a microtubule-governed distribution during mammalian cytokinesis.

Mitochondria are transported to the plus-ends of microtubules by kinesin motor proteins. Several studies have shown that Kinesin-1 is the primary motor for anterograde mitochondrial transport on microtubules [47], [48]. However, members of the Kinesin-3 subfamily, KIF1b and KLP6, have also been implicated in mitochondrial trafficking [49], [50]. In the case of Kinesin-1, binding to mitochondria is indirect and is mediated by the Miro-Milton complex [51], [52]. Miro has been shown to regulate the distribution of mitochondria during embryonic cell division in *Arabidopsis* [53]. The *Drosophila* kinesin-like protein, KLP67A, is essential for proper spindle assembly and, furthermore, when KLP67A activity is knocked down in *Drosophila* DL2 cells, mitochondria are mislocalized around the spindle [54], [55]. Whether kinesin motors play a role in recruiting mitochondria to the cleavage furrow in mammalian cytokinesis is an area for future work.

An important outcome of cytokinesis is the accurate inheritance of subcellular organelles. Indeed, other organelles including the endosomes, lysosomes, and Golgi are inherited by daughter cells via ordered mechanisms that are mediated by the mitotic spindle and the centrosomes [42]–[45]. Currently, the segregation of the mitochondria into daughter cells is assumed to be a stochastic process [56]. It has been shown that the activity of dynamin-related protein 1 (Drp1) is upregulated in early mitosis leading to extensive fragmentation of the mitochondrial network [22]. The fragmented mitochondria are then randomly dispersed throughout the cytoplasm, which is thought to facilitate stochastic inheritance [22]. In contrast, we demonstrate that mitochondria are specifically recruited to the cleavage furrow by the microtubule cytoskeleton, indicating that mitochondrial inheritance is not stochastic but that mitochondria are partitioned by an ordered, microtubule-mediated mechanism. An ordered mechanism may help to ensure the accuracy of mitochondrial partitioning between daughter cells or may allow the dividing cell to select for a specific subpopulation of mitochondria. In support of this, when microtubules were disrupted with Nocodazole and Taxol, mitochondrial inheritance by daughter cells showed increased variability (Figure 6C). However, since microtubule-disrupting drugs also perturb cytokinesis, further work is required to determine whether the increased variability of inheritance is specifically due to the inhibition of mitochondrial recruitment to the furrow.

The transport of mitochondria to meet local energy needs is important in highly differentiated and polarized cells. In particular, mitochondria are enriched in synapses and growth cones in neurons [57]–[61], immunological synapses [62]–[64], at the trailing edge in migrating lymphocytes [65] and surrounding the sperm axoneme [66]. Even in small epithelial cells of the kidney, mitochondria are predominantly localized to the basolateral surface in close proximity to membrane ATPases [67]. In addition, mitochondria have been shown to redistribute in response to changes in cellular physiology. For example, mitochondria translocate to the plasma membrane during calcium-dependent T-cell activation where they function to sustain the calcium signal [68]. Recently, it has been shown that mitochondria accumulate around the nucleus in arterial endothelial cells under hypoxic conditions, leading to an increase of ROS in the nucleus and induction of transcription [69]. During cytokinesis, the cell becomes highly polarized [70] and may, therefore, have specific energetic and metabolic requirements that would necessitate the local enrichment of mitochondria at the cleavage furrow. For example, ATP produced by mitochondria is required for the assembly and contraction of the cytokinetic ring [71]–[74] and is utilized by kinases at the cleavage furrow and kinesin motors in the spindle [74]–[79]. In addition, mitochondria act as highly localized buffers of calcium [68], [80]–[83] and localized calcium transients along the cleavage furrow have been observed in a number of embryonic divisions [84]–[86]. Global calcium transients have also been reported in dividing mammalian cells [87]. An under-appreciated function of mitochondria is the production of the membrane lipid, phosphatidylethanolamine (PE) [88]–[90]. It has been reported that PE is required for contractile ring disassembly and cytokinesis completion in mammalian cells [91]–[94]. The export of PE to other membranes requires close membrane contacts, which may explain why mitochondria are enriched at the furrow. Finally, mitochondria can govern local concentrations of ROS second messengers that participate in redox signaling [95], [96]. Certain ROS are short-lived and must be produced by mitochondria in close proximity to their site of action [97]. Whether mitochondria are recruited to the cleavage furrow to perform any of the aforementioned functions is currently unknown.

## Conclusion

In this study, we have shown that mitochondria are recruited to the cleavage furrow and away from the cell poles during cytokinesis in mammalian cells using a microtubule-dependent mechanism. The positioning of mitochondria by microtubules may help to ensure the accurate partitioning of mitochondria into daughter cells during cytokinesis. Alternatively, the recruitment of mitochondria to the cleavage furrow may facilitate cytokinesis by locally enriching mitochondrial activity. Further investigation is required to establish the molecular mechanism and functional significance of mitochondrial recruitment to the cleavage furrow in cytokinesis.

## Supporting Information

Figure S1 **Quantification of MitoTracker Green staining in dividing HeLa cells for comparison with MitoTracker Red, related to** **Figure 1** **.** Spinning disk confocal time-lapse images of HeLa cells stained with 40 nM MitoTracker Green to visualize mitochondria independently of mitochondrial membrane potential. Shown are a single focal plane from the center of the confocal stack (upper row) and the merge of the DIC image with the mitochondrial single focal plane (bottom row). Yellow arrowheads indicate the position of the cleavage furrow. Time is given in minutes after anaphase onset. Bar, 10 µm. (B) Quantification of the distribution of mitochondria from cell pole to equator at each stage of division. An overlay of all five stages is also shown (last panel). The normalized distance from cell pole to equator is displayed on the x-axis and the average mitochondrial fluorescence intensity is displayed on the y-axis. Data are represented as mean +/- SEM (14 cells, N = 56) and lines fitted by non-linear regression.
(TIF)

Figure S2 **Evaluation of actin and microtubule drug-treatments, related to** **Figures 5** **and** **6** **.** Top row: cells were treated for 15 min with either 0.1% DMSO (control), 100 nM Latrunculin A or 500 nM Jasplakinolide then fixed and stained for actin with Phalloidin 488. Yellow arrowheads indicate representative cells in which the majority of actin filaments have been depolymerized. Red arrowheads indicate representative cells with stabilized actin and increased focal adhesions. Bottom row: cells were treated for 5 min with 0.1% DMSO (control), 20 µM Nocodazole or 10 µM Taxol, then fixed and stained for microtubules (anti-alpha tubulin). Yellow arrowheads indicate representative cells in which the dynamic microtubule filaments have been depolymerized and only stable microtubules remain. Red arrowheads indicate cells with stabilized microtubules, which have similar staining to control cells but are not dynamic (for discussion see main text). Bar, 20 µm.
(TIF)

Figure S3 **Quantification of mitochondrial fluorescence intensity in actin drug-treated cells, related to** **Figure 5** **.** The normalized distance from cell pole to equator is displayed on the x-axis and the average mitochondrial fluorescence intensity is displayed on the y-axis. Data are represented as mean +/- SEM and lines fitted by non-linear regression.
(TIFF)

Figure S4 **Quantification of mitochondrial fluorescence intensity in microtubule drug-treated cells, related to** **Figure 6** **.** The normalized distance from cell pole to equator is displayed on the x-axis and the average mitochondrial fluorescence intensity is displayed on the y-axis. Data are represented as mean +/- SEM and lines fitted by non-linear regression.
(TIFF)

Movie S1 **Full time-series for** **Figure 1** **, showing mitochondria localized to the cleavage furrow in a dividing HeLa cell.** Images were acquired every 1 minute and the display rate is 3 frames / sec. Bar, 10 µm.
(MOV)

Movie S2 **Full time-series for** **Figure 4B** **, showing mitochondria localized to the cleavage furrow during monopolar cytokinesis.** Also shown is a second cell in which the cytokinetic cleavage is clearly visible. Red arrows indicate the position of the cleavage furrow. Images were acquired every 1 minute and the display rate is 3 frames / sec. Bar, 10 µm.
(MOV)

Movie S3 **Full time-series for** **Figure 5** **, showing mitochondria localized to the cell equator in Latrunculin A- and Jasplakinolide-treated cells.** Also shown is a DMSO-treated control cell for comparison. Images were acquired every 1 minute and the display rate is 3 frames / sec. Bar, 10 µm.
(MOV)

Movie S4 **Full time-series for** **Figure 6** **, showing mislocalized mitochondria in Nocodazole- and Taxol-treated cells.** Also shown is a DMSO-treated control cell for comparison. Red asterisks indicate the time of drug addition. Images were acquired every 1 minute and the display rate is 3 frames / sec. Bar, 10 µm.
(MOV)
