# Supplementary figures and images for: Mitochondria Localize to the Cleavage Furrow in Mammalian Cytokinesis
Source: PLoS One. 2013 Aug 21;8(8):e72886. doi: 10.1371/journal.pone.0072886 (PMC3749163; doi:10.1371/journal.pone.0072886)

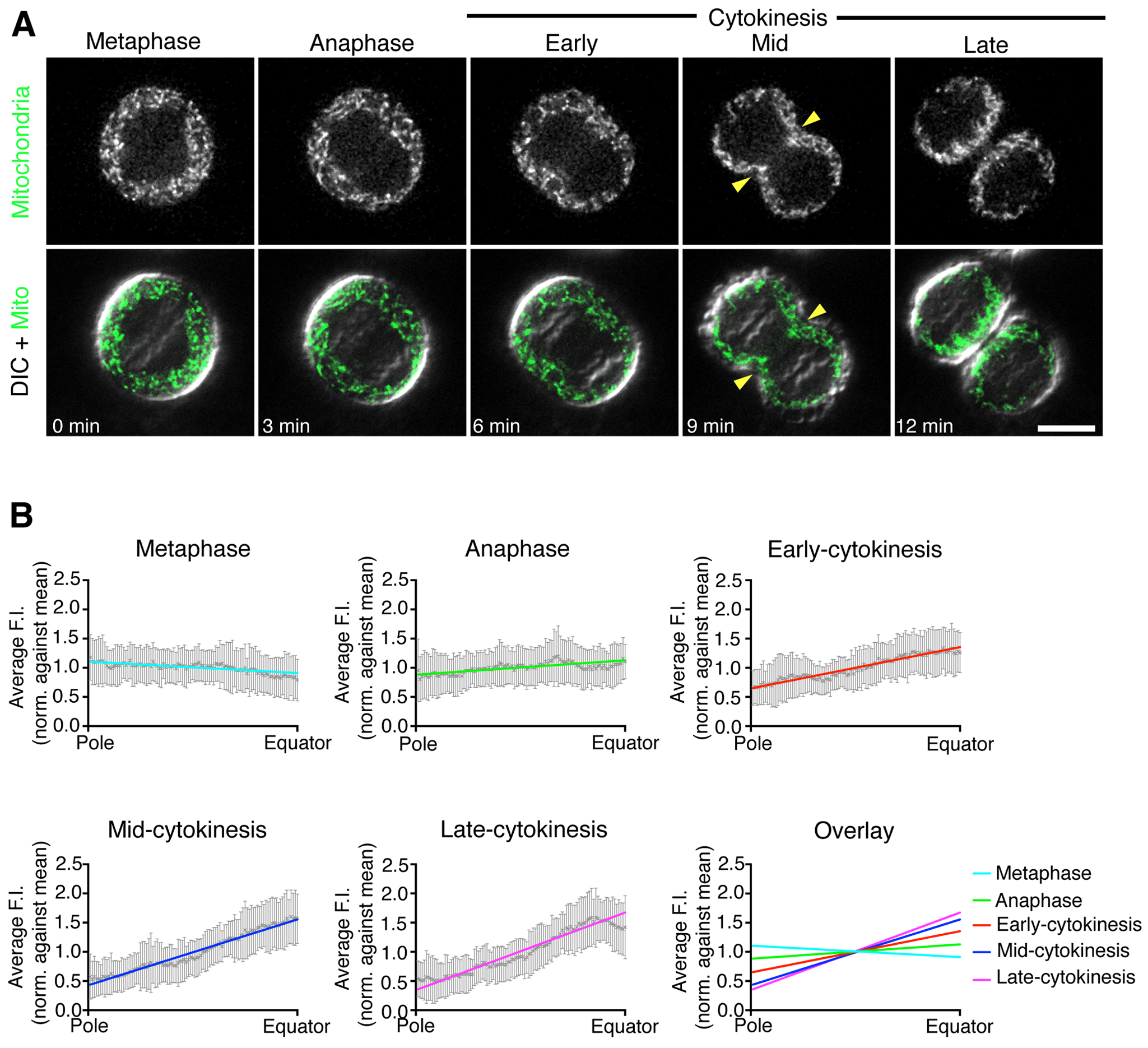

Supplement: Figure S1 — Quantification of MitoTracker Green staining in dividing HeLa cells for comparison with MitoTracker Red, related to Figure 1 . Spinning disk confocal time-lapse images of HeLa cells stained with 40 nM MitoTracker Green to visualize mitochondria independently of mitochondrial membrane potential. Shown are a single focal plane from the center of the confocal stack (upper row) and the merge of the DIC image with the mitochondrial single focal plane (bottom row). Yellow arrowheads indicate the position of the cleavage furrow. Time is given in minutes after anaphase onset. Bar, 10 µm. (B) Quantification of the distribution of mitochondria from cell pole to equator at each stage of division. An overlay of all five stages is also shown (last panel). The normalized distance from cell pole to equator is displayed on the x-axis and the average mitochondrial fluorescence intensity is displayed on the y-axis. Data are represented as mean +/- SEM (14 cells, N = 56) and lines fitted by non-linear regression. (TIF) [file pone.0072886.s001.tif]

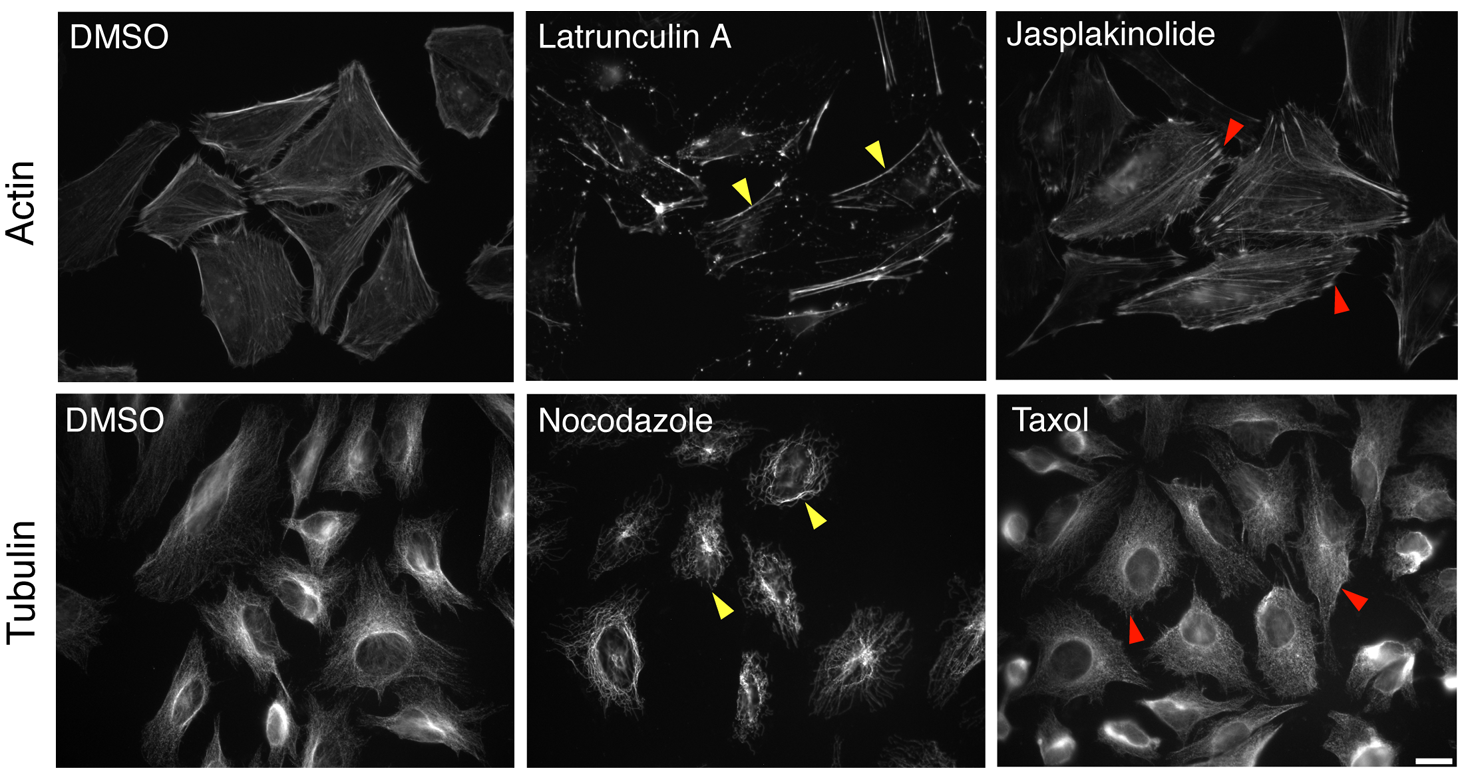

Supplement: Figure S2 — Evaluation of actin and microtubule drug-treatments, related to Figures 5 and 6 . Top row: cells were treated for 15 min with either 0.1% DMSO (control), 100 nM Latrunculin A or 500 nM Jasplakinolide then fixed and stained for actin with Phalloidin 488. Yellow arrowheads indicate representative cells in which the majority of actin filaments have been depolymerized. Red arrowheads indicate representative cells with stabilized actin and increased focal adhesions. Bottom row: cells were treated for 5 min with 0.1% DMSO (control), 20 µM Nocodazole or 10 µM Taxol, then fixed and stained for microtubules (anti-alpha tubulin). Yellow arrowheads indicate representative cells in which the dynamic microtubule filaments have been depolymerized and only stable microtubules remain. Red arrowheads indicate cells with stabilized microtubules, which have similar staining to control cells but are not dynamic (for discussion see main text). Bar, 20 µm. (TIF) [file pone.0072886.s002.tif]

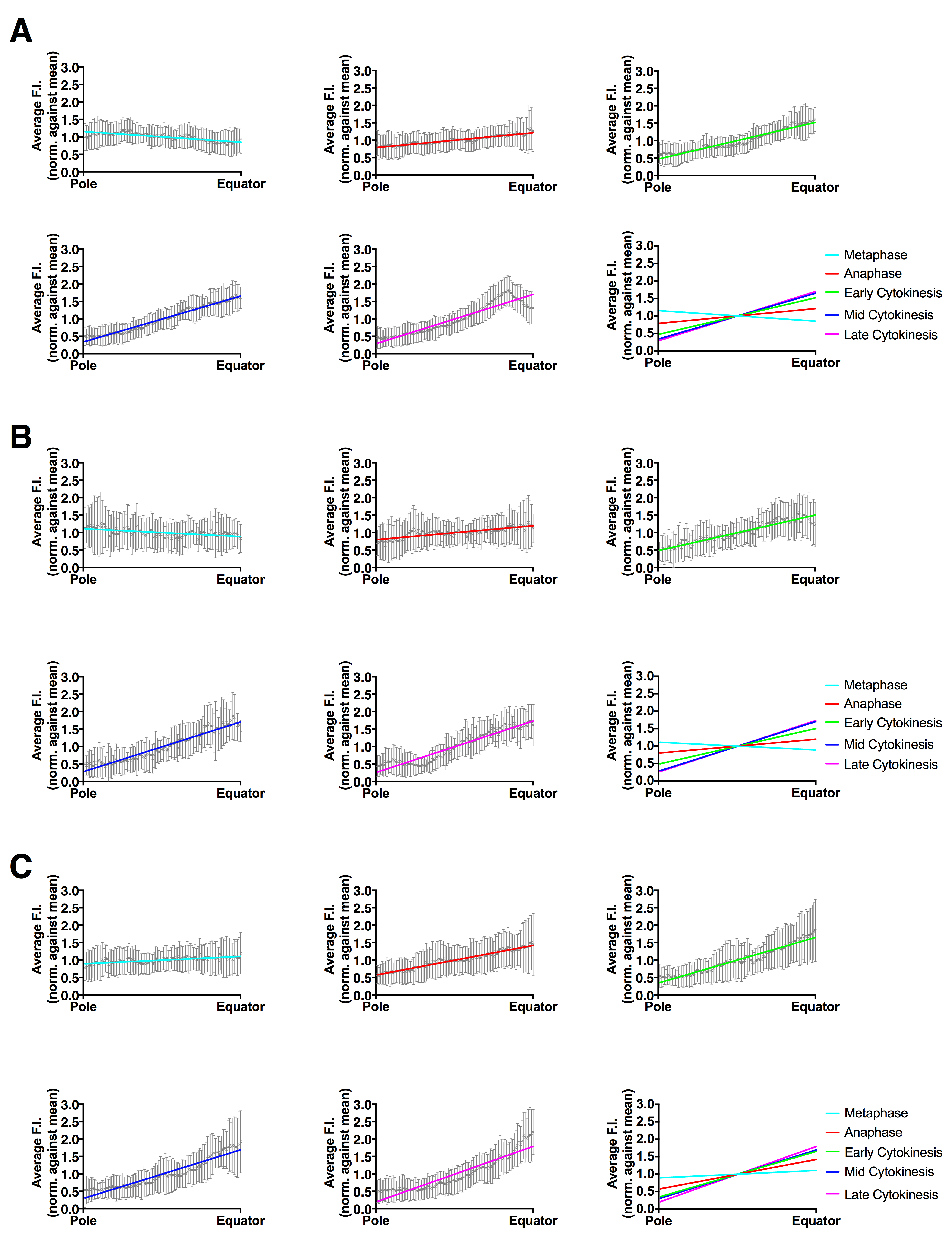

Supplement: Figure S3 — Quantification of mitochondrial fluorescence intensity in actin drug-treated cells, related to Figure 5 . The normalized distance from cell pole to equator is displayed on the x-axis and the average mitochondrial fluorescence intensity is displayed on the y-axis. Data are represented as mean +/- SEM and lines fitted by non-linear regression. (TIFF) [file pone.0072886.s003.tiff]

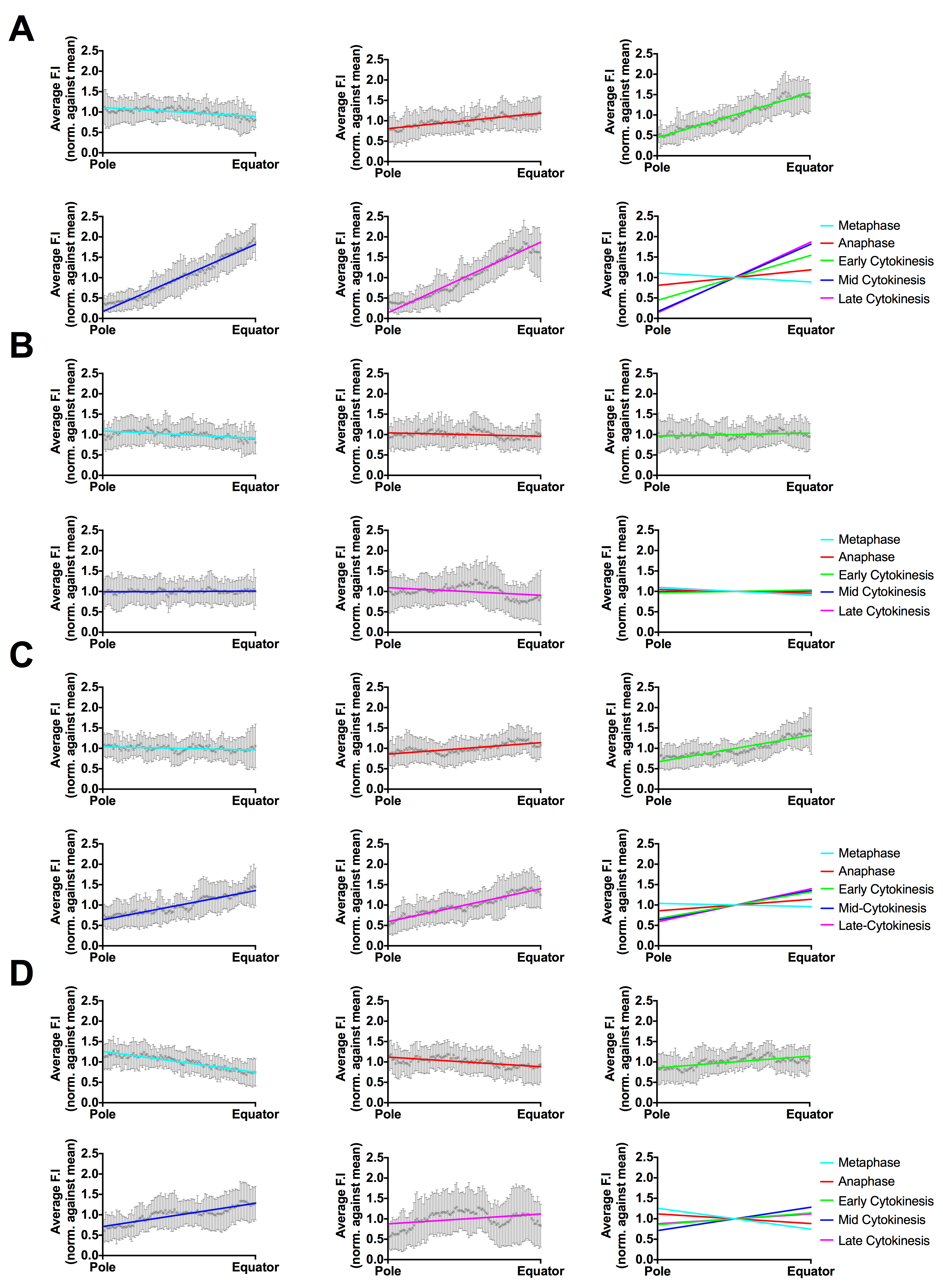

Supplement: Figure S4 — Quantification of mitochondrial fluorescence intensity in microtubule drug-treated cells, related to Figure 6 . The normalized distance from cell pole to equator is displayed on the x-axis and the average mitochondrial fluorescence intensity is displayed on the y-axis. Data are represented as mean +/- SEM and lines fitted by non-linear regression. (TIFF) [file pone.0072886.s004.tiff]
